# Electron Bridge Enhanced Charge Polarization Enables Efficient and Ultra‐Long Stable Urea Electrosynthesis

**DOI:** 10.1002/anie.2196422

**Published:** 2026-09-15

**Authors:** Fan Wu, Haixia Wang, Wei Wang, Junrong Zou, Ruijie Zhou, Fenglin Xie, Peng Gao, Weiliu Fan, Nana Wang, Zhongchao Bai, Guoxiu Wang, Wei Ye

**Affiliations:** ^1^ Key Laboratory of Organosilicon Chemistry and Material Technology, Ministry of Education, Zhejiang Key Laboratory of Organosilicon Material Technology, College of Material Chemistry and Chemical Engineering Hangzhou Normal University Hangzhou Zhejiang P. R. China; ^2^ School of Chemistry and Chemical Engineering Shandong University Jinan P. R. China; ^3^ Centre For Clean Energy Technology School of Mathematical and Physical Sciences Faculty of Science University of Technology Sydney Sydney NSW Australia; ^4^ University of Shanghai for Science and Technology Shanghai P. R. China

**Keywords:** charge polarization, C–N coupling, dual single‐atom alloys, electron bridge, urea electrosynthesis

## Abstract

Urea electrosynthesis from carbon dioxide and nitrates powered by green electricity is a zero‐carbon route for urea production. Simultaneously achieving high activity and long‐term stability is a prerequisite for the industrialization of urea electrosynthesis. In this study, we propose an electron bridge strategy based on a Pd_1_ and Ni_1_ dual single‐atom alloy to boost the electrocatalytic activity and durability for urea electrosynthesis. The single‐atom Ni_1_ sites act as electron bridges to promote charge transfer from Cu to Pd_1_ sites, leading to charge‐polarized Pd–Cu sites, which upshift the d‐band center and improve oxidation resistance of the catalyst. Consequently, the Pd_1_ and Ni_1_ dual single‐atom sites simultaneously accelerates both carbon dioxide reduction and nitrates reduction half‐reactions, while facilitating the first C–N coupling step. The Pd_1_ and Ni_1_ dual single‐atom alloys deliver a urea yield rate of 541.6 mmol g_cat_^−1^ h^−1^ with a urea Faradaic efficiency of 57.3%. Remarkably, this catalyst achieves a record durability of 1700 h at a current density of 40 mA cm^−2^. This work provides new insights into the synergistic catalysis of complex multi‐molecule reactions through cooperative multiple active sites.

## Graphical abstract

An electron bridge strategy is developed to regulate charge polarization in Pd_1_ and Ni_1_ dual single‐atom sites, promoting CO_2_ reduction, nitrate reduction, and C–N coupling simultaneously. The resulting catalyst achieves efficient urea electrosynthesis with high activity and record durability over 1700 h.

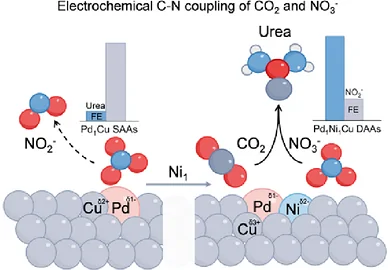

## 1 Introduction

Urea is the world's most demanded basic nitrogenous fertilizer and an important chemical feedstock, playing irreplaceable roles in agricultural production and materials manufacturing [1, 2, 3]. The annual global production of urea is approximately 120 million tons [1, 4, 5]. To date, industrial urea production still relies on the century‐old and energy‐intensive Bosch–Meiser process, in which carbon dioxide (CO_2_) and ammonia (NH_3_) are coupled under harsh conditions (temperature: ∼200°C, pressure: ∼210 bar) [3, 6]. The consumption of fossil fuels for urea production accounts for roughly 1%–2% of the global annual energy supply, resulting in severe carbon emissions [1, 7]. Direct urea synthesis from carbon dioxide and nitrates (NO_3_^−^) powered by green electricity has recently emerged as a promising zero‐carbon alternative to the conventional Bosch–Meiser process [7, 8, 9].

However, the practical application of urea electrosynthesis is still limited by low urea yield rates and Faradaic efficiencies (FEs), mainly originating from the difficulties in co‐activating CO_2_ and NO_3_^−^, the low C–N coupling efficiency, and the complex competing reaction pathways [8, 10, 11]. Moreover, the inherent oxidizing ability of nitrates makes the catalyst prone to oxidation during electrolysis, leading to unsatisfactory durability [12, 13]. As a result, the electrochemical co‐reduction of CO_2_ and NO_3_^−^ still falls short of the economic viability targets predicted by techno‐economic assessments. To improve electrochemical performance, efficient activation of both CO_2_ and NO_3_^−^ is essential. More importantly, the key C‐ and N‐containing intermediates generated in situ during CO_2_ reduction reaction (CO_2_RR) and nitrate reduction reaction (NO_3_RR) should preferentially undergo efficient C–N coupling rather than separate deep reduction pathways, which strongly depend on the adsorption strength of the intermediates [10, 14, 15]. Excessively strong adsorption tends to induce over reduction of the C‐/N‐intermediates, whereas overly weak adsorption leads to intermediate desorption from the catalyst surface before coupling occurs. Therefore, precisely regulating the electronic structure of the catalyst is crucial for optimizing the adsorption and activation of CO_2_ and NO_3_^−^, as well as to facilitate C–N coupling process [10, 16]. Charge polarization induced by intermetallic charge transfer redistributes the electron density to regulate the d‐band center, optimize intermediate adsorption, and simultaneously improve the oxidation resistance of catalysts [17]. However, achieving precise control over the degree of charge polarization to simultaneously balance catalytic activity and long‐term stability remains highly challenging.

Herein, a novel electron bridge strategy based on Pd_1_ and Ni_1_ dual single‐atom alloys (DAAs) is proposed to regulate charge polarization, thereby boosting the activity and durability of urea electrosynthesis. The single‐atom Ni_1_ sites act as electron bridges to enhance charge transfer between Pd and adjacent Cu sites, thereby improving the oxidation resistance of the Cu host. Meanwhile, the enhanced charge polarization optimizes the adsorption of *CO and *NH_2_ intermediates and promotes the first C–N coupling step toward urea formation. Moreover, the charge polarization also accelerates both CO_2_RR and NO_3_RR half‐reactions. The as‐synthesized Pd_1_Ni_1_Cu DAAs deliver a high urea yield rate of 541.6 mmol g_cat_^−1^ h^−1^ with a urea FE of 57.3% in a flow cell. Notably, the catalyst achieves outstanding operational stability over 1700 h, far exceeding the previously reported values.

## 2 Result and Discussion

PdNiCu ternary alloy catalysts were synthesized by a simple wet chemical reduction method using Pd^2+^, Ni^2+^, and Cu^2+^ as precursors and NaBH_4_ as the reducing agent [18]. To precisely tune the molar ratios of Pd:Ni:Cu in the alloys, the amounts of Pd^2+^ and Ni^2+^ precursors were adjusted while keeping the Cu amount constant. Inductively coupled plasma‐atomic emission spectroscopy (ICP‐AES, Table S1) confirms that the elemental ratios in the obtained samples are generally consistent with the feeding ratios. Accordingly, the alloy samples are labelled as Pd_x_Ni_y_Cu_5_, where x, y, and 5 represent the precursor molar ratios. Figure 1a shows the x‐ray diffraction (XRD) patterns of the alloy samples. The main diffraction peaks of Pd_1_Ni_1_Cu_5_ are located at 42.5°, 50.1°, and 74.2°, assigned to the (111), (200), and (220) crystal planes of the face‐centered cubic (*fcc*) structure, respectively. Notably, these diffraction peaks are located between those of *fcc* Pd, Ni, and Cu, confirming the formation of PdNiCu ternary alloy. Furthermore, as the Pd or Ni content gradually increases, the diffraction peaks of the Pd_x_Ni_y_Cu_5_ samples progressively shift toward those of Pd or Ni, consistent with the feature of alloy formation (Figure S1) [19, 20, 21, 22]. In contrast, both Pd_1_Cu_5_ and Ni_1_Cu_5_ samples exhibit an additional diffraction peak at 36.5°, assigning to the Cu_2+1_O (111) crystal plane, indicating partial surface oxidation of Cu upon air exposure. The co‐doping of Pd and Ni into the Cu lattice significantly suppresses the surface oxidation of Cu, which is likely associated with the charge transfer among Pd, Ni and Cu atoms [23, 24].

**FIGURE 1:**
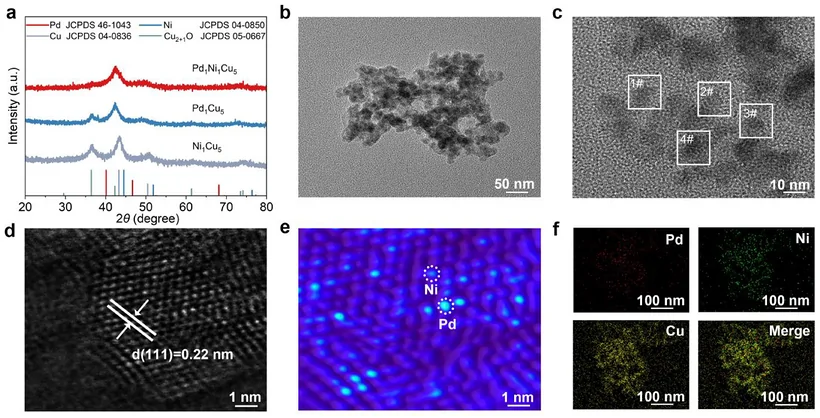
(a) XRD patterns of Pd_1_Ni_1_Cu_5_, Pd_1_Cu_5_, and Ni_1_Cu_5_ alloy samples. (b, c) TEM images, (d) atomic‐resolution HAADF‐STEM image, (e) three‐dimensional topographic atom image of Pd_1_Ni_1_Cu_5_ alloy. (f) Elemental mapping images of Pd_1_Ni_1_Cu_5_ sample, Pd, red; Ni, green; Cu, yellow.

Transmission electron microscopy (TEM) images reveal that the Pd_1_Ni_1_Cu_5_ sample is composed of randomly stacked small particles with an average particle size of ∼15.7 nm (Figures 1b,c, and S2). The atomic resolution high‐angle annular dark‐field scanning TEM (HAADF‐STEM) image shows clear two‐dimensional atomic matrix with a lattice distance of 0.22 nm, corresponding to the (111) plane of the ternary alloy (Figure 1d). The three‐dimensional topological atom image (Figure 1e) clearly reveals atomically dispersed Pd atoms with higher brightness and larger atomic radius within the Cu lattice. Furthermore, the Ni atoms, exhibiting similar size and brightness to the Cu host atoms, are also atomically dispersed. These results indicate the formation of Pd_1_ and Ni_1_ dual single‐atom alloys, denoted as Pd_1_Ni_1_Cu DAAs. Furthermore, EDS elemental mapping images (Figure 1f) demonstrate the uniform distribution of Pd, Ni, and Cu throughout the nanoparticles, further confirming the alloy structure. For comparison, Pd_1_Cu single‐atom alloys (SAAs, i.e., Pd_1_Cu_5_ sample) and Ni_1_Cu SAAs (i.e., Ni_1_Cu_5_ sample) samples were also synthesized and characterized (Figures S3–S6).

X‐ray photoelectron spectroscopy (XPS) measurement was employed to characterize the electronic structure of Pd_1_Ni_1_Cu DAAs sample (Figure S7). The high‐resolution Pd 3d spectrum in Figure 2a is deconvoluted into two sets of double peaks. The peaks located at 335.0 and 340.3 eV are assigned to 3d_5/2_ and 3d_3/2_ of Pd^0^, respectively. Another pair of peaks at 335.9 and 341.4 eV are attributed to 3d_5/2_ and 3d_3/2_ of Pd^2+^, respectively [23]. The Ni 2p spectrum (Figure 2b) shows the co‐existence of Ni^0^ (855.4, 873.2 eV) and Ni^2+^ (860.5, 878.2 eV) signal. The peaks at 863.9 and 881.4 eV are the satellite (Sat.) peaks of Ni^2+^ [25, 26]. Notably, compared with metallic Pd and Ni, the Pd^0^ peaks in Pd_1_Ni_1_Cu DAAs and Pd_1_Cu SAAs negatively shift by 0.52 and 0.18 eV, respectively. Similarly, Ni^0^ peaks also negatively shift 0.20 and 0.18 eV, respectively, indicating electron accumulation around Pd and Ni sites [17, 27]. Moreover, Ni doping further enhances the electron accumulation on Pd site, which is verified by the larger position shift in Pd_1_Ni_1_Cu DAAs. The charge polarization is further supported by the differential charge density map (Figures 2c and S8), where electron accumulation is mainly concentrated around Pd and Ni atoms in Pd_1_Ni_1_Cu DAAs.

**FIGURE 2:**
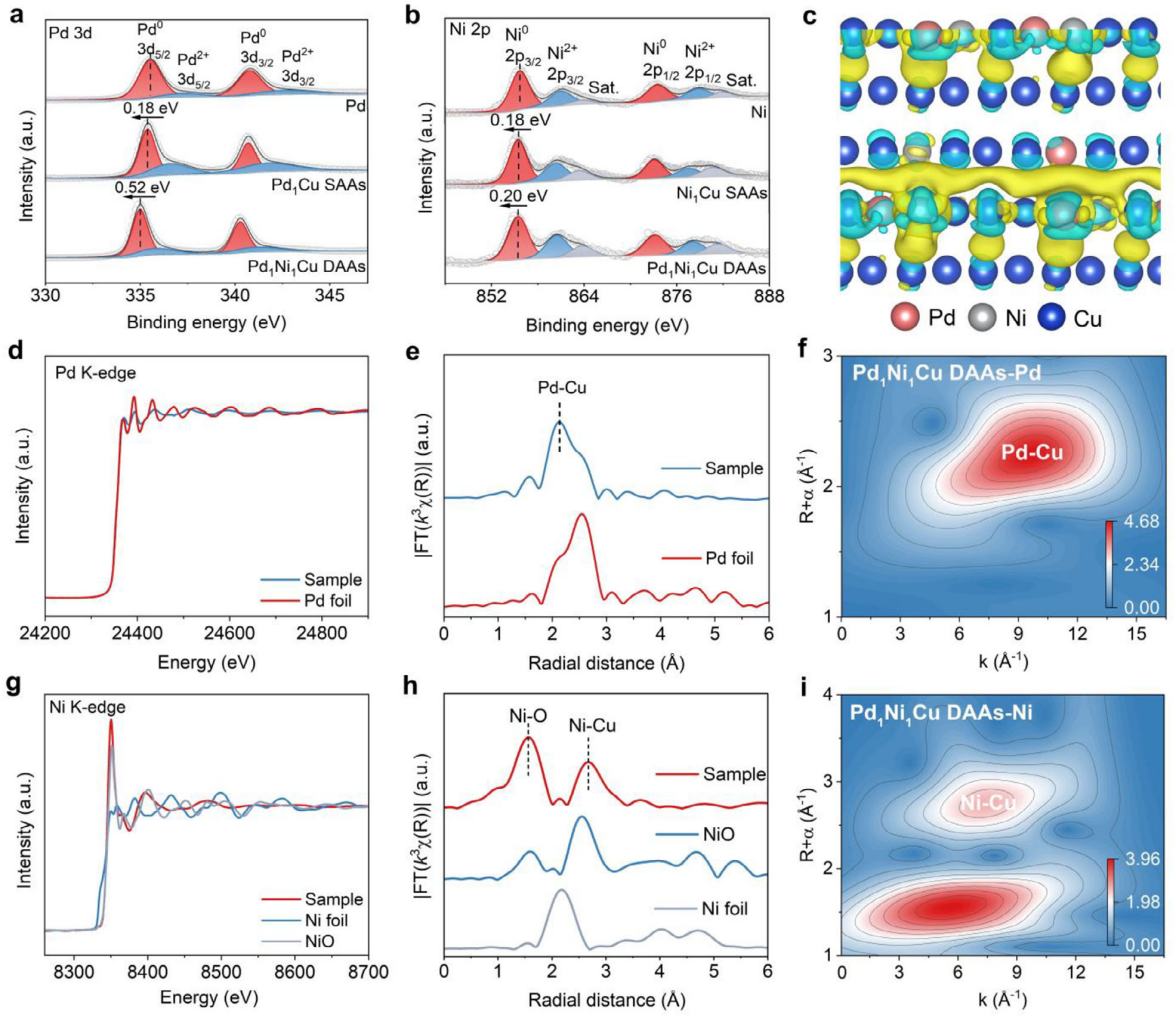
(a) Pd 3d spectrum, (b) Ni 2p spectrum, (c) differential charge density map of Pd_1_Ni_1_Cu DAAs. (d) Normalized Pd *K*‐edge XANES spectrum of Pd_1_Ni_1_Cu DAAs with Pd foil as reference. (e) *k* ^3^‐weighted Fourier‐transform EXAFS spectra. (f) Wavelet transform of the *k* ^3^‐weighted Pd *K*‐edge EXAFS signal. (g) Normalized Ni *K*‐edge XANES spectrum of Pd_1_Ni_1_Cu DAAs with Ni foil and NiO as reference samples. (h) *k* ^3^‐weighted Fourier‐transform Ni *K*‐edge EXAFS spectra. (i) Wavelet transform of the *k* ^3^‐weighted Ni *K*‐edge EXAFS signal.

To further resolve the local coordination environment and atomic structure of the alloy, x‐ray absorption fine structure (XAFS) spectroscopy measurements were performed. Figure 2d shows the Pd K‐edge x‐ray absorption near‐edge structure (XANES) spectrum of the Pd_1_Ni_1_Cu DAAs sample with Pd foil as the reference. The XANES spectrum of Pd_1_Ni_1_Cu DAAs nearly overlaps with that of Pd foil, suggesting the metallic state of Pd in the alloy. The corresponding extended XAFS (EXAFS) spectrum only reveals Pd─Cu bonds with bond length of 2.72 Å and coordination number (CN) of 7.3, while no Pd─Pd bonds, confirming the single‐atom Pd_1_ dispersion in the alloy (Figures 2e and S9a; Table S2) [27, 28, 29]. The Wavelet transform, together with the fitting results in *R* space and *k* space, further confirms only Pd─Cu bonds in the sample and no Pd─Pd bonds (Figures 2f and S10a–S13a) [27]. Figure 2g,h shows the Ni *K*‐edge XANES and EXAFS spectra of the Pd_1_Ni_1_Cu DAAs sample, respectively. The absorption edge of Ni in the sample is closer to that of NiO, featuring the oxidation state of Ni in the sample. Similarly, the EXAFS spectrum only resolves Ni─Cu and Ni─O bonds in the first coordination shell, with bond lengths of 2.54 and 1.96, and CNs of 6.7 and 3.1, respectively. No Ni─Ni bonds are resolved in the alloy sample, also manifesting the atomic dispersion of Ni_1_ in the alloy. The Wavelet transform and the fitting results in *R* space and *k* space (Figures 2i and S9b–S13b) confirm the fine structure of single‐atom Ni_1_ in the alloy [26]. Combined with the HAADF‐STEM results, the XAFS analyses confirm the dual single‐atom dispersion of Pd_1_ and Ni_1_ within the Cu host lattice, namely Pd_1_Ni_1_Cu DAAs.

The urea electrosynthesis performance of the alloy samples was evaluated in an H‐type cell using a three‐electrode configuration, in which the anode and cathode chambers were separated by a commercial Nafion 117 membrane. The urea produced in the catholyte was spectrophotometrically quantified using the diacetyl monoxime colorimetric method and the urease decomposition method (Figures S14 and S15). Liquid by‐products such as NO_2_^−^ were quantified using the Griess reagent colorimetric method (Figure S16), while gaseous by‐products (e.g., CO and H_2_) were quantified by gas chromatography (GC, Figure S17) [15, 27, 30, 31]. To evaluate the electrocatalytic response of the Pd_1_Ni_1_Cu DAAs, linear sweep voltammetry (LSV) test was first conducted in different electrolytes. As shown in Figure 3a, the current densities follow the order of *I*(KNO_3_ + KHCO_3_) > *I*(KNO_3_ + KHCO_3_ + CO_2_) > *I*(KNO_3_) > *I*(KHCO_3_ + CO_2_). Obviously, the current response in the system containing both CO_2_, HCO_3_^−^, and NO_3_^−^ is significantly higher than the individual NO_3_RR (tested in KNO_3_ solution) and CO_2_RR (tested in KHCO_3_ solution with CO_2_ bubbling), suggesting the Pd_1_Ni_1_Cu DAAs may deliver good urea electrosynthesis performance [32].

**FIGURE 3:**
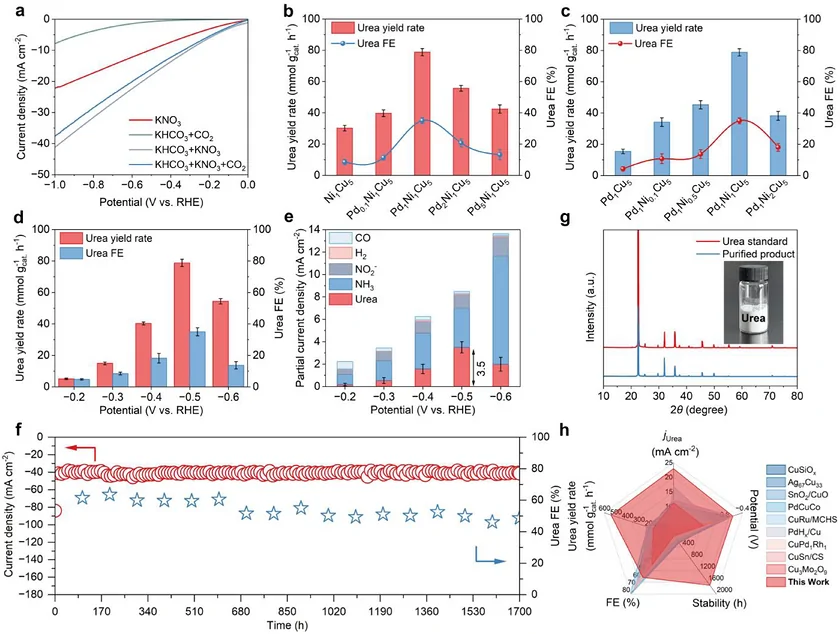
(a) LSV curves of Pd_1_Ni_1_Cu DAAs recorded in different electrolytes. (b, c) The comparison of urea electrosynthesis performance of Pd_x_Ni_y_Cu_5_ samples with different Pd and Ni doping level assessed at −0.5 V in a mixture of 0.1 M KHCO_3_ + 0.1 M KNO_3_ with CO_2_ bubbling. (d) Potential dependence of urea yield rates and urea FEs of Pd_1_Ni_1_Cu DAAs in H‐type cell, (e) the corresponding partial current densities of all the possible products. (f) Ultralong‐term *I*–*t* curve of Pd_1_Ni_1_Cu DAAs operated in the flow cell at −0.5 V. (g) XRD pattern of the urea powder collected from the cathodic electrolyte after the durability test. (h) Electrocatalytic performance comparison of Pd_1_Ni_1_Cu DAAs with the recently reported Cu‐based electrocatalysts.

Next, the Pd and Ni doping levels in the ternary alloy (Pd_x_Ni_y_Cu_5_) as well as the concentrations of nitrates were systematically optimized to achieve the best urea electrosynthesis performance (Figure S18). As shown in Figure 3b, both the urea yield rates and FEs of the Pd_x_Ni_1_Cu_5_ alloys exhibit a volcano‐type dependence on the Pd content. A similar trend is also observed when tuning the Ni content in the Pd_1_Ni_y_Cu_5_ alloys (Figure 3c). Among all the samples, Pd_1_Ni_1_Cu_5_, namely Pd_1_Ni_1_Cu DAAs, delivers the optimal urea electrosynthesis performance. Then, the potential dependence of urea electrocatalytic performance of the Pd_1_Ni_1_Cu DAAs was further assessed (Figure S19). As shown in Figure 3d, the urea production activities at the applied potentials of −0.2, −0.3, −0.4, −0.5, and −0.6 V are 5.1, 14.9, 40.3, 78.8, and 54.4 mmol g_cat_^−1^ h^−1^, respectively. Correspondingly, the urea FEs are 4.8%, 8.5%, 18.2%, 35.0%, and 13.7%. Control experiments demonstrate that urea is only generated when CO_2_ and NO_3_^−^ coexist in the electrolyte, confirming that the urea formation originates from the C–N coupling process between CO_2_ and NO_3_^−^ (Figure S20) [28, 31, 32, 33]. In addition, ^15^N and ^13^C isotope labeling experiments further confirm that the nitrogen and carbon sources of urea are nitrate ions and CO_2_ (Figures S21 and S22) [28, 32, 33]. The urea quantification results were further verified by ^1^H NMR spectroscopy, and the comparable results further confirm the reliability of the measurements (Figures S23 and S24) [27, 31].

To better understand the product selectivity during co‐electrolysis, the partial current densities (*j*) and FEs of the possible products in the co‐electrolysis were calculated. The *j*_urea_ rapidly increases with increasing cathodic potential and reaches a maximum value of 3.5 mA cm^−2^ at −0.5 V (Figure 3e). The partial current density of NH_3_ (*j*_NH3_), as the major by‐product, exhibits a similar trend. However, at −0.6 V, the *j*_NH3_ sharply increases while the *j*_CO_ (the key C‐intermediate for C–N coupling) remains nearly unchanged. The kinetic mismatch between NO_3_RR and CO_2_RR under strongly negative potentials leads to a decrease in urea FEs [27]. In contrast, *j*_H2_ and *j*_NO2−_ remain relatively stable over the tested potential range. Accordingly, the variation trend of NH_3_ FEs is consistent with that of *j*_NH3_, while the NO_2_^−^ FEs remain below 20% throughout the entire potential window (Figure S25).

For comparison, the urea electrosynthesis performance of Pd_1_Cu SAAs and Ni_1_Cu SAAs was also evaluated (Figures S19a,b, S26, and S27). The optimal urea yield rates of Pd_1_Cu SAAs and Ni_1_Cu SAAs are 15.5 and 30.2 mmol g_cat_^−1^ h^−1^, with corresponding urea FEs of 4.3% and 8.5%, respectively. Notably, the optimal urea FE of the Pd_1_Ni_1_Cu DAAs sample are 8.1 and 4.1 times higher than those of Pd_1_Cu SAAs and Ni_1_Cu SAAs, respectively, indicating the significantly enhanced C–N coupling efficiency due to the co‐doping of Pd_1_ and Ni_1_ dual single‐atom within the Cu lattice (Figure S28). Cyclic voltammetry (CV) tests show that Pd_1_Ni_1_Cu DAAs deliver a larger electrochemically active surface area (ECSA) than both Pd_1_Cu SAAs and Ni_1_Cu SAAs (Figure S29). Moreover, electrochemical impedance spectroscopy (EIS, Figure S30) test also shows that Pd_1_Ni_1_Cu DAAs exhibit a much lower charge transfer resistance. These results suggest that the incorporation of Pd_1_ and Ni_1_ dual single‐atoms not only increase the ECSA, but also facilitates charge transfer, thereby enhancing the electrocatalytic performance [34].

Long‐term operational stability is another critical parameter for evaluating electrocatalysts. Repeated electrolysis tests in H‐type cell demonstrate that Pd_1_Ni_1_Cu DAAs maintain stable urea yield rates and FEs over 20 consecutive cycles (20 h) without noticeable performance decay (Figure S31). After the durability test, the catalyst was further characterized by XRD, TEM, HRTEM, EDS elemental mapping and XPS measurements (Figures S32–S35). The phase structure, the morphology, the elemental distribution and valence states remain well preserved, confirming the rigidity of the dual single‐atom alloy catalyst.

Considering the excellent catalytic activity and stability of the Pd_1_Ni_1_Cu DAAs, the urea electrosynthesis performance was further assessed in a flow cell equipped with a gas diffusion electrode (GDE) to overcome the bottleneck of the low mass transfer kinetics of CO_2_. As expected, the current density at −0.5 V in GDE greatly increases to 40 mA cm^−2^ (Figure 3f). Accordingly, the average urea yield rate sharply increases to 541.6 mmol g_cat_^−1^ h^−1^, accompanied by an improved urea FE of 57.3% due to the meliorative mass transfer of CO_2_ gas [27]. Remarkably, Pd_1_Ni_1_Cu DAAs delivers an excellent stability over 1700 h at −0.5 V in GDE (Figure 3f), far exceeding the previously reported values. In contrast, the metallic Cu catalyst displays gradual decay of urea performance, mainly attributed to the chemical oxidation of metallic Cu to Cu_2_O (Figure S37) [35]. The result indicates that the co‐doping of Pd_1_ and Ni_1_ dual single‐atom strengthens the antioxidant capacity of Cu host. After the ultralong‐term electrolysis, the produced urea in the catholyte was extracted, and finally 3.40 g of highly pure urea powder was obtained, which is also confirmed by the XRD measurement (Figure 3g). Notably, Pd_1_Ni_1_Cu DAAs significantly outperform the current state‐of‐the‐art Cu‐based electrocatalysts in terms of urea yield rates, *j*_urea_, and durability (Figure 3h; Tables S3 and S4) [32, 36, 37, 38, 39, 40, 41, 42, 43].

Upon evaluating the electrochemical performance, it is essential to elucidate the specific role of Pd_1_ and Ni_1_ dual single‐atom in Cu lattice on enhancing C–N coupling process. Typically, the co‐electrolysis of CO_2_ and NO_3_^−^ toward urea formation is a complex multi‐step reaction involving 16 electrons and 18 protons, accompanied by multiple C/N‐intermediates and various possible C–N coupling pathways [27, 28]. To better understand the reaction mechanism, the electrocatalytic performances of the individual half‐reactions, that is, CO_2_RR and NO_3_RR, were first investigated to reveal the respective roles of Pd_1_ and Ni_1_ sites in C/N‐related intermediates conversion processes. Figure 4a,b compare the *j*_CO_ and *j*_NH3_ values of Pd_1_Ni_1_Cu DAAs, Pd_1_Cu SAAs, and Ni_1_Cu SAAs in the independent CO_2_RR and NO_3_RR processes, respectively (Figures S38 and S39). As the applied potential negatively shifts, the *j*_CO_ of Pd_1_Cu SAAs is consistently higher than Ni_1_Cu SAAs, indicating that the PdCu sites are more active for CO_2_RR‐to‐CO conversion than NiCu sites. In contrast, Ni_1_Cu SAAs deliver higher *j*_NH3_ value than Pd_1_Cu SAAs, verifying the NiCu sites are more favorable for nitrate‐to‐ammonia conversion than PdCu sites. Pd_1_Ni_1_Cu DAAs deliver both the highest *j*_CO_ and *j*_NH3_ but relatively lower *j*_CO/ECSA_ and *j*_NH3/ECSA_ normalized to ECSA, demonstrating that the co‐doping of Pd_1_ and Ni_1_ strengthens CO_2_RR and NO_3_RR half‐reactions by improving ECSA (Figures S29 and S40).

**FIGURE 4:**
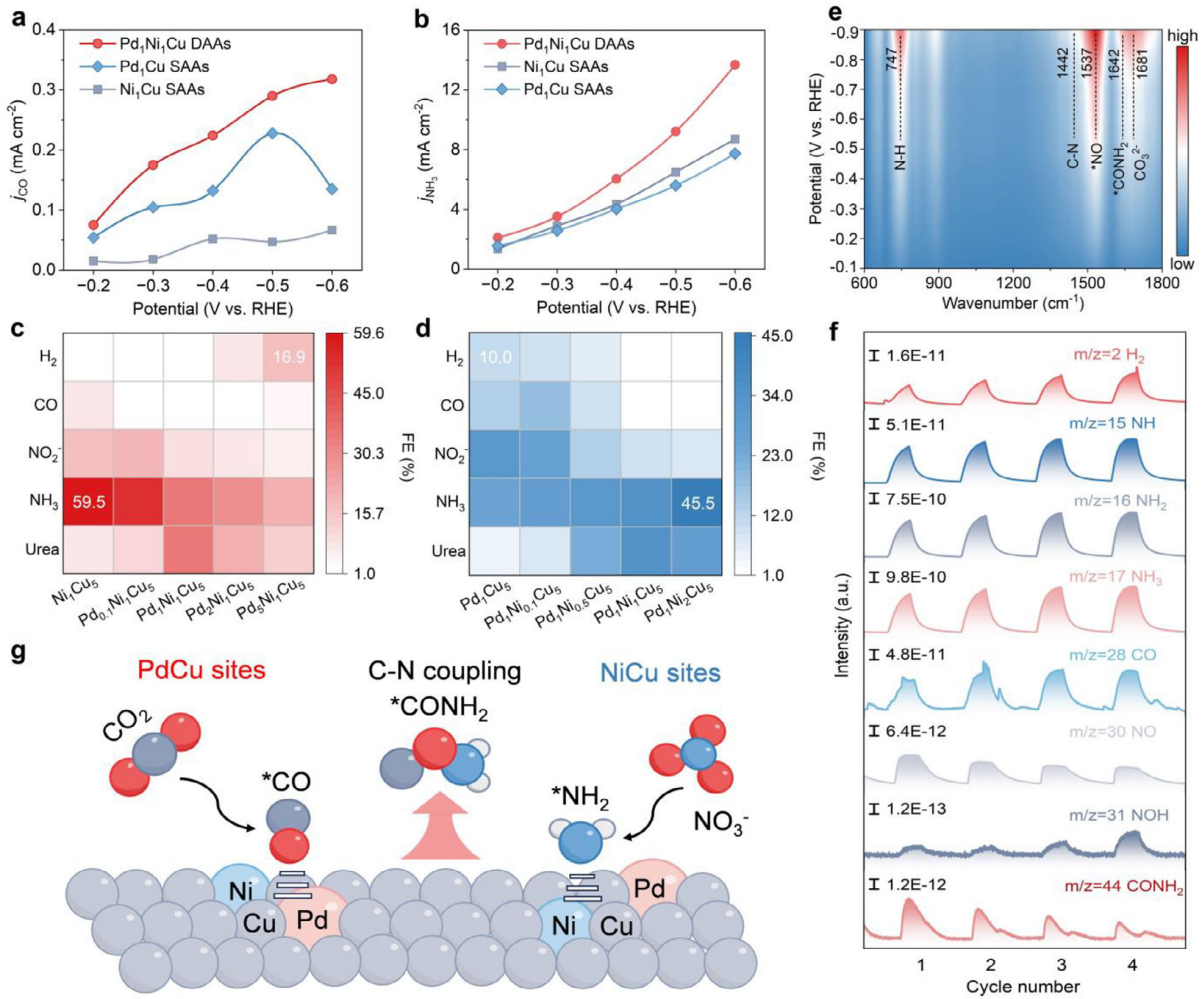
Potential dependence of (a) *j*_CO_ in sole CO_2_RR and (b) *j*_NH3_ in sole NO_3_RR of Pd_1_Ni_1_Cu DAAs, Ni_1_Cu SAAs, and Pd_1_Cu SAAs. Potential dependence of the FEs of the possible products in urea electrosynthesis over (c) Pd_x_Ni_1_Cu_5_ and (d) Pd_1_Ni_y_Cu_5_ alloys samples with different Pd and Ni doping level. (e) Potential dependence in situ ATR‐FTIR spectra of Pd_1_Ni_1_Cu DAAs between −0.1 and −0.9 V with an interval of 0.1 V. (f) Online DEMS spectra of Pd_1_Ni_1_Cu DAAs in urea electrosynthesis. (g) Schematic illustration of the synergistic catalytic mechanism of Pd_1_Ni_1_Cu DAAs for C–N coupling.

To further investigate the effect of Pd_1_ and Ni_1_ dual single‐atom sites on C–N coupling, the corresponding FEs of the major products for Pd_x_Ni_1_Cu_5_ and Pd_1_Ni_y_Cu_5_ alloys were analyzed. As shown in Figure 4c, the NH_3_ FE dominates in the Ni_1_Cu_5_ alloy system, reaching 59.5%, revealing that Ni_1_Cu SAAs strongly facilitates NH_3_ formation during co‐electrolysis. With increasing Pd doping level, both NO_2_^−^ and NH_3_ FEs gradually decrease, while the urea FE continuously increases. These results suggest two important points: first, the co‐doping of Pd and Ni effectively promotes the NO_2_^−^‐to‐NH_3_ conversion process; second, the accelerated generation kinetics of N‐intermediates facilitates C–N coupling rather than simply favoring NH_3_ formation. However, when the Pd content is further increased, the CO FE rises sharply while the urea FE decreases, further confirming that PdCu sites strongly promote CO_2_RR to CO conversion. Figure 4d further shows the relationship between possible products FEs and the Ni doping level in Pd_1_Ni_y_Cu_5_ alloy. For the Pd_1_Cu_5_ alloy, the NO_2_^−^ FE is much higher than NH_3_ FE, indicating sluggish NO_2_^−^‐to‐NH_3_ conversion on Pd_1_Cu_5_ alloy. As the Ni doping level gradually increases, the NO_2_^−^ FE continuously decreases while the NH_3_ FE correspondingly increases, manifesting the promotion role of Ni doping for NO_2_^−^‐to‐NH_3_ conversion. In addition, the volcano‐plot variation of urea FEs with increasing Ni content indicates that the excessive Ni doping results in overly fast N‐intermediates formation kinetics, leading to mismatched C/N‐reduction kinetics and consequently reduced C–N coupling efficiency. Beyond that, Ni doping in the ternary alloy significantly decreases the H_2_ FEs, mainly because the accelerated NO_3_RR process on Pd_1_Ni_1_Cu_5_ alloy consumes a large amount of active hydrogen species (*H), thereby inhibiting the coupling of *H to generate H_2_ [44].

In situ attenuated total reflection Fourier transform infrared (ATR‐FTIR) spectroscopy characterization provides the most direct molecular‐level evidence to infer the C–N coupling pathway [45]. As the potential negatively shifts from −0.1 to −0.9 V, the vibration peak at 1537 cm^−1^ gradually intensifies, attributing to the *NO intermediate (Figure 4e) [46]. Subsequently, a clear N–H bending vibration signal arises at 747 cm^−1^, assigning to the *NH_2_ species from NO_3_RR [46]. Meanwhile, the peak at 1442 cm^−1^ is assigned to the characteristic C–N stretching vibration, and the *CONH_2_ signal located at 1642 cm^−1^ simultaneously emerges and continuously strengthens, suggesting that urea formation proceeds through the *CO + *NH_2_→*CONH_2_ pathway [47].

To further capture the reaction intermediates during co‐electrolysis, online differential electrochemical mass spectrometry (DEMS) was further carried out. Intermediates signal corresponding to H_2_ (*m*/*z* = 2), NH (*m*/*z* = 15), NH_2_ (*m*/*z* = 16), NH_3_ (*m*/*z* = 17), CO (*m*/*z* = 28), NO (*m*/*z* = 30), NOH (*m*/*z* = 31), and CONH_2_ (*m*/*z* = 44) are detected (Figure 4f). In particular, the CO signal confirms the CO_2_RR‐to‐CO conversion process, while the detection of the key CONH_2_ intermediate directly supports the first C–N coupling pathway of *CO + *NH_2_→*CONH_2_, which is also verified by the control experiments in Table S5 and consistent with the in situ ATR‐FTIR result [14, 27, 48, 49]. Combined with the *j*_CO_ and *j*_NH3_ results from the half‐reactions, as well as FE evolution trends during the co‐electrolysis, these results demonstrate that the dual single‐atom Pd_1_ and Ni_1_ in Cu lattice synergistically promotes both the CO_2_RR‐to‐*CO and NO_3_^−^‐to‐*NH_2_ processes, while simultaneously facilitating the coupling of *CO and *NH_2_ toward urea formation (Figure S41) [50]. The proposed mechanism is illustrated in Figure 4g.

To deeply investigate the promotion effect of Pd_1_ and Ni_1_ dual single‐atom sites on C–N coupling, density functional theory (DFT) calculations were carried out. In the theoretical model, Cu atoms on the Cu(111) crystal plane are partially replaced by Pd_1_ and Ni_1_ atoms to construct dual single‐atom alloy structure. Notably, the possible Ni─O bonds in the catalyst were ignored for simplicity as the actual condition was operated at negative potential region and most of Ni─O species could be reduced. The electron localization function (ELF) images (Figures 5a and S42; Table S6) indicate that the Pd_1_ sites in Pd_1_Ni_1_Cu DAAs exhibit higher electron accumulation than those in Pd_1_Cu SAAs. Bader charge analysis further reveals that the isolated Pd_1_ and Ni_1_ atoms obtain −1.15 e^−^ and −0.16 e^−^, respectively, while the adjacent Cu atoms lose 0.30 e^−^, resulting in the atomically polarized Pd^δ1−^Cu^δ3+^ and Ni^δ2−^−Cu^δ3+^ sites. Notably, both the ELF results, Bader charge analysis and differential charge density analysis indicate that the charge polarization between Pd and Cu atoms is further enhanced after Ni_1_ doping (Figure S43). The result confirms that the Ni_1_ atom acts as the electron bridge to facilitate charge transfer from Cu to Pd_1_ sites. Accordingly, the charge polarization redistributes the charge density of the Pd_1_ and Ni_1_ single‐atom sites [51]. As revealed by the partial density of states (PDOS) calculations (Figure S44), the d‐band center of Pd_1_Ni_1_Cu DAAs upshifts to −1.61 eV compared with Pd_1_Cu SAAs (−1.94 eV) and Ni_1_Cu SAAs (−1.87 eV). Notably, the d‐band center closer to the Fermi level generally leads to stronger interactions between the catalytic sites and reaction intermediates [52].

**FIGURE 5:**
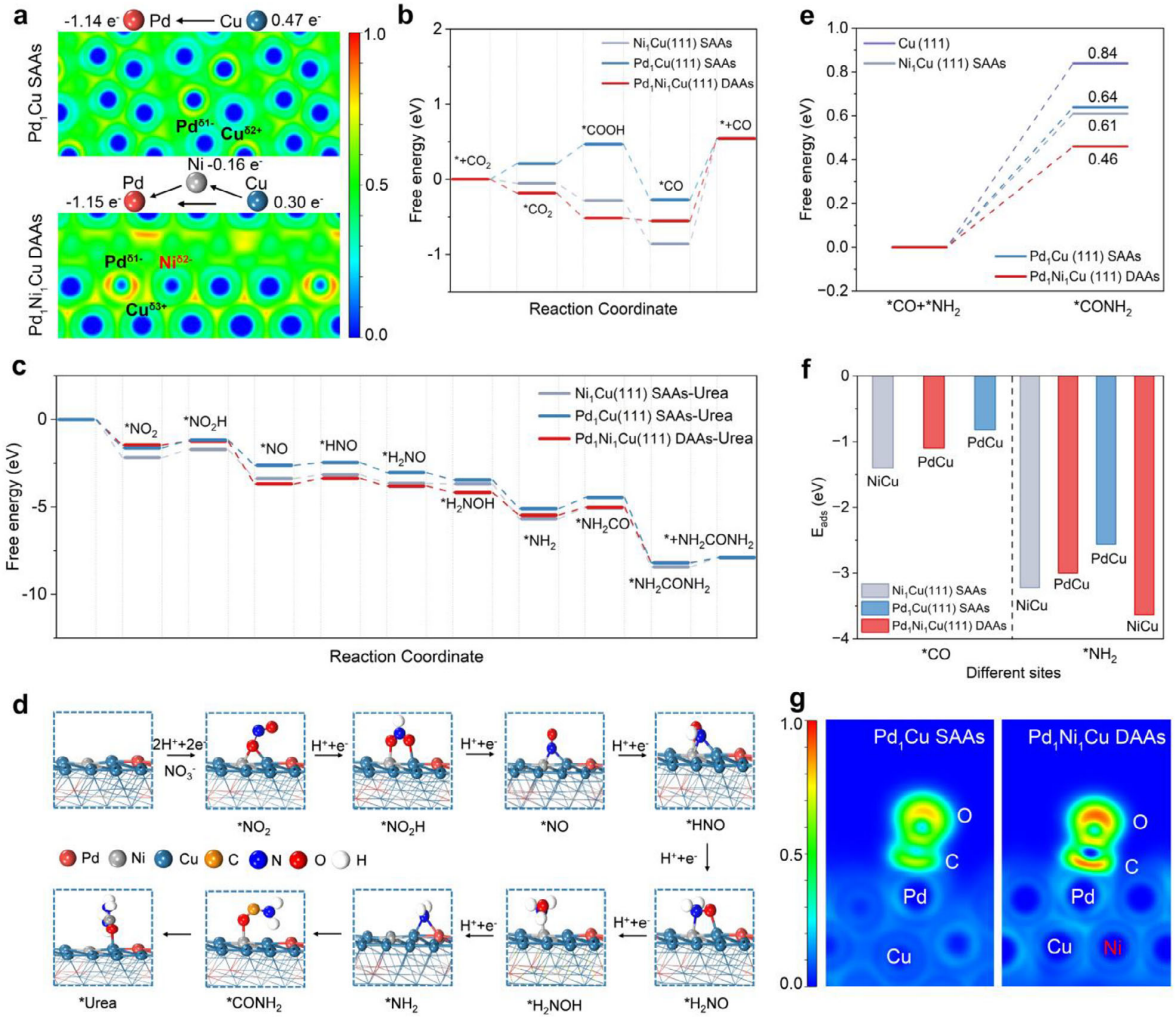
(a) ELF pictures of Pd_1_Ni_1_Cu DAAs and Pd_1_Cu SAAs. (b) Energy profiles of each elementary step in single CO_2_RR catalyzed on Pd_1_Cu(111) SAAs, Ni_1_Cu(111) SAAs, and Pd_1_Ni_1_Cu(111) DAAs planes. (c) Energy profiles of each elementary step in urea synthesis catalyzed by Pd_1_Cu(111) SAAs, Ni_1_Cu(111) SAAs, and Pd_1_Ni_1_Cu(111) DAAs planes. (d) DFT‐calculated urea formation cycle on Pd_1_Ni_1_Cu(111) DAAs surface. (e) The calculated energy barrier of the PDS (the 1^#^ C–N coupling) in urea formation with *NH_2_ adsorbed on different active sites. (f) The comparison of the adsorption energy of the *CO and *NH_2_ on different sites. (g) ELF picture of the *CO on Pd_1_Ni_1_Cu DAAs and Pd_1_Cu SAAs.

To decode the impact of the enhanced charge polarization on urea formation, the free energy profiles of CO_2_RR and urea synthesis on Pd_1_Ni_1_Cu(111) DAAs, Pd_1_Cu(111) SAAs, and Ni_1_Cu(111) SAAs were calculated. For CO_2_RR on Ni_1_Cu(111) SAAs (Figure 5b), all elementary steps involved in the CO_2_‐to‐CO conversion are thermodynamically downhill, while the *CO desorption from the catalyst surface requires overcoming an energy barrier of 1.40 eV, which is regarded as the potential‐determining step (PDS). In comparison, on Pd_1_Cu(111) SAAs, both CO_2_ adsorption and the *CO_2_→*COOH conversion are endothermic, whereas the energy barrier for *CO desorption is significantly reduced to 0.82 eV, explaining the improved CO evolution capability of Pd_1_Cu SAAs. Similar to Ni_1_Cu SAAs, the conversion from CO_2_ to *CO on Pd_1_Ni_1_Cu DAAs (PdCu sites) is thermodynamically favorable, while the energy barrier of the PDS lies between those of Pd_1_Cu SAAs and Ni_1_Cu SAAs. These results indicate that Pd_1_Ni_1_Cu DAAs can simultaneously promote both CO_2_ to *CO conversion and *CO desorption, leading to the highest CO generation rate among the three catalysts.

Figure 5c shows the free‐energy profiles of urea formation on Pd_1_Ni_1_Cu(111) DAAs, Pd_1_Cu(111) SAAs, and Ni_1_Cu(111) SAAs, while the corresponding adsorption configurations are demonstrated in Figures 5d, S45, and S46. Notably, for Pd_1_Ni_1_Cu DAAs, urea formation and/or NO_3_RR initially proceed on NiCu sites until the formation of *H_2_NO. Subsequently, the intermediates dynamically transfer among Ni_1_, PdCu, Ni_1_ and Cu_1_ sites to minimize the reaction energy barrier during the conversion process. From the free‐energy analysis, the *NO_2_→*NO_2_H, *NO→*HNO, *NH_2_→*NH_2_CO, and urea desorption steps are endothermic, while the remaining elementary steps are exothermic. Among them, the 1^#^ C–N coupling step is identified as the PDS for all three catalysts. The energy barrier of this step on Pd_1_Ni_1_Cu(111) DAAs is calculated to be 0.46 eV, which is lower than those on Ni_1_Cu(111) SAAs (0.61 eV), Pd_1_Cu(111) SAAs (0.64 eV) and metallic Cu(111) (0.84 eV) (Figure 5e). Based on the calculated energy barriers and adsorption configurations, the synergistic effect of Pd_1_ and Ni_1_ dual single‐atom sites can be summarized as follows: (1) the intermediates can dynamically hop among different sites to follow the energetically favorable pathway; (2) The regulated electronic structure lowers the energy barrier of key C–N coupling step.

The adsorption strength of *CO and *NH_2_, that is, the key C‐/N‐intermediates for the 1^#^ C–N coupling, significantly affects the coupling efficiency. Generally, both excessively strong and excessively weak adsorption are unfavorable for efficient C–N coupling [53]. Hence, the adsorption energy of *CO and *NH_2_ on different sites was calculated. As shown in Figure 5f, adsorption strength of both *NH_2_ and *CO on PdCu sites of Pd_1_Ni_1_Cu(111) DAAs lies between those on NiCu sites of Ni_1_Cu(111) SAAs and PdCu sites of Pd_1_Cu(111) SAAs, suggesting more moderate adsorption behavior on Pd_1_Ni_1_Cu(111) DAAs. This optimized adsorption originates from the enhanced charge polarization induced by the Ni_1_ electron bridge. For the key C‐intermediate of *CO, the enhanced polarization of PdCu sites allows more electrons to be injected into the π antibonding orbital of *CO, resulting in stronger CO adsorption. This conclusion is further supported by the ELF images of *CO adsorbed on Pd_1_Ni_1_Cu DAAs and Pd_1_Cu SAAs. As expected, more electron density is concentrated around *CO adsorbed on Pd_1_Ni_1_Cu(111) DAAs than on Pd_1_Cu SAAs (Figures 5g and S47), further confirming that the enhanced charge polarization indeed facilitates the adsorption and activation of the key C‐/N‐intermediates.

## 3 Conclusion

In summary, a tunable atomical charge polarization strategy was developed through the co‐doping of Pd_1_ and Ni_1_ dual single‐atom in Cu lattice to synergistically promote the NO_3_RR, CO_2_RR and C–N coupling toward efficient urea electrosynthesis. Single‐atom Ni_1_ sites serve as the electron bridge to enhance the charge transfer from Cu to Pd_1_ sites. The regulated electronic structure up‐shifts the d‐band center and enables moderate adsorption of the key *CO and *NH_2_ intermediates, thereby facilitating the C–N coupling process. Beyond that, the enhanced charge polarization effectively suppresses the oxidation of the Cu host. Meanwhile, the co‐doping of Pd_1_ and Ni_1_ dual single‐atom also effectively accelerates both CO_2_RR and NO_3_RR kinetics. Benefitting from the promoted CO_2_RR, NO_3_RR and C–N coupling processes, the Pd_1_Ni_1_Cu DAAs deliver a high urea yield rate of 541.6 mmol g_cat_^−1^ h^−1^ with a urea FE of 57.3%. More importantly, the alloy catalyst achieves a record durability and can stably operate in GDE over 1700 h at a current density of 40 mA cm^−2^, far exceeding the currently reported copper‐based catalysts. This work provides new insights for designing highly active and durable alloy catalysts for C–N coupling from the angle of atomic‐level charge polarization.

## Author Contributions

**Fan Wu**: conceptualization, writing – original draft, methodology, formal analysis. **Haixia Wang**: methodology. **Wei Wang**: methodology. **Junrong Zou**: methodology. **Ruijie Zhou**: methodology. **Fenglin Xie**: methodology. **Peng Gao**: writing – review and editing. **Weiliu Fan**: methodology, writing – review and editing. **Nana Wang**: methodology, writing – review and editing. **Zhongchao Bai**: methodology. **Guoxiu Wang**: writing – review and editing, methodology. **Wei Ye**: conceptualization, methodology, writing – review and editing, supervision, formal analysis.

## Conflicts of Interest

The authors declare no conflicts of interest.

## Supporting information




**Supporting File**: anie74869‐sup‐0001‐SuppMat.docx.

## Data Availability

The data that supports the findings of this study are available in the Supporting Information of this article.
