# Supplementary material for: Electron Bridge Enhanced Charge Polarization Enables Efficient and Ultra‐Long Stable Urea Electrosynthesis
Source: Angew Chem Int Ed Engl. 2026 Sep 15;65(45):e2196422. doi: 10.1002/anie.2196422 (PMC13725571; doi:10.1002/anie.2196422)
Supplement: Supplementary file 1 [file ANIE-65-e2196422-s001.docx]

**Electron Bridge Enhanced Charge Polarization Enables Efficient and Ultra-long Stable Urea Electrosynthesis**

Fan Wu,^a,+^ Haixia Wang,^b,+^ Wei Wang,^a,+^ Junrong Zou,^a^ Ruijie Zhou,^a^ Fenglin Xie,^a^ Peng Gao,^a^ Weiliu Fan,^b,^* Nana Wang,^c,^* Zhongchao Bai,^d^ Guoxiu Wang^c,^* and Wei Ye^a,^*

^a^ Key Laboratory of Organosilicon Chemistry and Material Technology, Ministry of Education, Zhejiang Key Laboratory of Organosilicon Material Technology, College of Material, Chemistry and Chemical Engineering, Hangzhou Normal University, Hangzhou, Zhejiang 311121, P. R. China.

^b^ School of Chemistry and Chemical Engineering, Shandong University, Jinan 250100, P. R. China.

^c^ Centre for Clean Energy Technology, School of Mathematical and Physical Sciences, Faculty of Science, University of Technology Sydney, Sydney, NSW, 2007 Australia.

^d^ University of Shanghai for Science and Technology, Shanghai, 200093, P. R. China.

^+^ These authors contributed equally to this work.

**Experimental Procedures**

**Chemicals.**

Potassium palladium(II) chloride (K_2_PdCl_4_, A.R.), copper(II) chloride dihydrate (CuCl_2_‧2H_2_O, A.R.), nickel chloride hexahydrate (NiCl_2_‧6H_2_O, A.R.), urea (CH_4_N_2_O, A.R.), sodium salicylate (NaC_7_H_5_O_3_, A.R.), monopotassium monosodium tartrate tetrahydrate (KNaC_4_H_12_O_10_, A.R.), sodium hydroxide (NaOH, A.R.), sodium hypochlorite (NaClO, A.R.), sodium nitroprusside (FeNa_2_C_5_H_4_N_6_O_3_, A.R.), ammonium chloride (NH_4_Cl, A.R.), sodium nitrite (NaNO_2_, A.R.), ethanol (EtOH, A.R.), ^15^N-labeled potassium nitrate (K^15^NO_3_, ≥99.5%), potassium nitrate (KNO_3_, A.R.), potassium bicarbonate (KHCO_3_, A.R.), ultrahigh purity CO_2_ (99.999%), ultra-high purity Ar (99.999%) and Griess reagent were purchased from Sinopharm Chemical Reagent Co. Ltd. (Shanghai, China). All chemicals were used without further purification. All aqueous solutions were prepared using de-ionized (DI) water with a resistivity of 18.25 MΩ‧cm^–1^.

**Methods.**

**Synthesis of Pd_1_Ni_1_Cu DAAs**

K_2_PdCl_4_ (0.1 mmol), NiCl_2_‧6H_2_O (0.1 mmol) and CuCl_2_·2H_2_O (0.5 mmol) were dissolved in deionized water (20 mL). After ultrasonic treatment for 10 minutes, the NaBH_4_ solution (10 mM, 10 mL) cooled with ice water was added in the above solution. After stirred gently for 30 minutes, the sample was collected by centrifugation and washed with water/ethanol for three times, respectively. Finally, the sample was vacuum-dried overnight. The protocol for the synthesis of Pd_x_Ni_y_Cu_5_ alloy samples was similar except with different amounts of Pd^2+^ and Ni^2+^ precursors.

**Synthesis of Pd_1_Cu SAAs**

K_2_PdCl_4_ (0.1 mmol) and CuCl_2_·2H_2_O (0.5 mmol) were dissolved in deionized water (20 mL). After ultrasonic treatment for 10 minutes, the NaBH_4_ solution (10 mM, 10 mL) cooled with ice water was added in the above solution. After stirred gently for 30 minutes, the sample was collected by centrifugation and washed with water/ethanol for three times, respectively. Finally, the sample was vacuum-dried overnight.

**Synthesis of Ni_1_Cu SAAs**

NiCl_2_‧6H_2_O (0.1 mmol) and CuCl_2_·2H_2_O (0.5 mmol) were dissolved in deionized water (20 mL). After ultrasonic treatment for 10 minutes, the NaBH_4_ solution (10 mM, 10 mL) cooled with ice water was added in the above solution. After stirred gently for 30 minutes, the sample was collected by centrifugation and washed with water/ethanol for three times, respectively. Finally, the sample was vacuum-dried overnight.

**Sample characterizations**

Prior to the electron microscopic examinations, the nanostructure suspension was drop-casted onto a carbon-coated molybdenum grid and dried at room temperature. Transmission electron microscopy (TEM) and high-resolution TEM (HRTEM) images, along with the corresponding energy-dispersive X-ray spectroscopy (EDS) mapping profiles, were collected on a JEOL JEM-2100F field-emission high-resolution transmission electron microscope operating at an accelerating voltage of 200 kV. Microstructural analysis was performed using an aberration-corrected scanning transmission electron microscope (JEM ARM 200F, Japan) equipped with a cold field-emission gun operating at 200 kV. Powder X-ray diffraction (XRD) patterns were recorded on a Philips X’Pert Pro Super diffractometer utilizing Cu-K radiation (λ=1.5418 A˚). X-ray photoelectron spectroscopy (XPS) measurements were conducted on an ESCALab 250 X-ray photoelectron spectrometer using a non-monochromatized Al-K_α_ X-ray source. The Pd and Ni K-edge X-ray absorption fine structure (XAFS) measurements were carried out at the 14W1 beamline of the Shanghai Synchrotron Radiation Facility (SSRF), China. The storage ring was operated at a typical energy of 2.5 GeV with a maximum current of 250 mA, and the hard X-rays were monochromatized using a Si (111) double-crystal monochromator.

**Electrosynthesis of urea in H-cell**

Specifically, 2 mg of the Pd_1_Ni_1_Cu DAAs was dispersed in a mixture containing 0.7 mL of deionized water, 0.25 mL of isopropanol, and 50 μL of Nafion solution (5 wt%), followed by ultrasonication for 30 minutes to form a homogeneous catalyst ink. Subsequently, 50 μL of the catalyst ink was uniformly drop-casted onto carbon paper with a defined area of 1×1 cm^2^ (catalyst loading: 0.1 mg cm^–2^). After drying under an infrared lamp, the resulting product served as the working electrode. An Ag/AgCl electrode and a Pt mesh electrode were employed as the reference and counter electrodes, respectively. The electrochemical tests were performed at room temperature in a typical three-electrode configuration, where the cathodic and anodic compartments were separated by a commercial Nafion 117 membrane. The electrolyte used for both the cathode and anode was a mixed solution of KHCO_3_ (40 mL, 0.1 M) and KNO_3_ (0.1 M). Prior to the electrochemical measurements, the electrolyte solution was continuously purged with ultra-high-purity CO_2_ gas (99.999%) for 30 minutes to reach CO_2_ saturation. The urea electrosynthesis was conducted at constant applied potentials (–0.2 V, –0.3 V, –0.4 V, –0.5 V, and –0.6 V) under a continuous flow of CO_2_. All applied potentials were converted to the reversible hydrogen electrode (RHE) scale according to the following Nernst equation:

*E*(*vs*. RHE) = *E*(*vs.* Ag/AgCl) + 0.198 V + 0.059 V × pH	(1)

The collected liquid products were subjected to quantitative analysis for urea using both the diacetyl monoxime method and the urease decomposition method. Possible liquid by-products in the electrolyte (e.g., ammonia and nitrite ions) were spectrophotometrically quantified using the indophenol blue method and the Griess reagent, respectively. Potential gaseous by-products (e.g., carbon monoxide and hydrogen) were quantitatively analyzed via gas chromatography (GC). The electrochemical performance of other samples was evaluated using identical procedures, merely by substituting the working electrode materials.

**Electrosynthesis of urea in flow cells**

The long-term durability test was performed using a commercial flow cell equipped with gas diffusion electrode. The volumes of the cathode and anode compartment are all 1×1×0.2 cm^3^. The distance of cathode and anode electrodes is 0.4 cm. To prepare the catalyst ink for the flow cell, 2 mg of the Pd_1_Ni_1_Cu DAAs was ultrasonically dispersed in a mixture comprising 0.7 mL of deionized water, 0.25 mL of isopropanol, and 50 μL of Nafion (5 wt%) for 30 minutes. Then, the catalyst ink was slowly drop-casted onto a gas diffusion layer (GDL) with catalyst loading amount of 0.1 mg cm^–2^. After drying under an infrared lamp, it was employed as the working electrode. An Ag/AgCl electrode and a Pt foil were used as the reference and counter electrodes, respectively. The catholyte (2 L) is a mixture of KNO_3_ (0.1 M) and KOH (1 M), while the anolyte was a KOH (1 M, 2 L) solution. CO_2_ gas (flow rate of 15 sccm) was continuously pumped into the system, and the flow rate of the electrolyte was 5 mL min^–1^ both for cathode and anode. Once the flow cell was successfully assembled and operated stably, urea electrosynthesis was triggered at –0.5 V versus RHE. To avoid the significant decline of NO_3_^–^ concentration and keep the pH stable, the cathode electrolyte was replaced with a fresh one every 200 h. The urea in the electrolyte solution was quantitatively analyzed every 100 hours using the diacetyl monoxime and the urease decomposition method.

**Determination of urea**

**Method 1:** Ethylenediaminetetraacetic acid (EDTA, 0.1 g) was dissolved in a urease solution (10 mL, 5 mg mL^–1^). Subsequently, 0.2 mL of the solution was added to the post-electrolysis electrolyte (1.8 mL) to reach a total volume of 2 mL. The final mixture was incubated and reacted in a shaker at 37 ℃ for 40 minutes. The generated ammonia was then spectrophotometrically quantified using the indophenol blue method.

**Method 2:** The urea generated in the electrolyte was spectrophotometrically quantified using the diacetyl monoxime (DAMO) method. Typically, 1 mL of the electrolyte was mixed with 2 mL of an acidic iron solution (prepared by mixing 100 mL of concentrated phosphoric acid, 300 mL of concentrated sulfuric acid, 600 mL of deionized water, and 100 mg of ferric chloride). Then,1 mL of a DAMO-thiosemicarbazide (TSC) solution (prepared by dissolving 5 g of DAMO and 100 mg of TSC in 1000 mL of deionized water) was added to the mixture. Following this, the solution was heated to 100℃ and maintained at this temperature for 20 minutes. After cooling down to room temperature, the absorbance was measured at 525 nm. A series of standard urea solutions were used to plot the calibration curve for urea determination.

**Determination of ammonia (NH_3_)**

Sodium salicylate (5 g) and sodium potassium tartrate (Seignette salt, 5 g) were dissolved in a NaOH solution (100 mL, 1 M) to obtain solution A. Sodium hypochlorite (3.5 mL,10%-15%) was diluted with 96.5 mL of deionized water to obtain solution B. Sodium nitroprusside (0.2 g) was dissolved in 20 mL of deionized water to obtain solution C. Solution A, solution B, and solution C were sequentially added to the diluted electrolyte solution (2 mL). After incubating the mixture in the dark at room temperature for 2 hours, its absorbance was measured at 655 nm. A series of standard NH_3_ solutions were utilized to establish the calibration curve for NH_3_ quantification.

**Determination of nitrite ions (NO_2_^–^)**

Nitrite ions (NO_2_^–^) were quantitatively determined via spectrophotometry using the Griess reagent. Typically, 200 μL of the Griess reagent was added to the electrolyte solution (5 mL). The mixed solution was then heated to 100 ℃ and held at this temperature for 1 minute. After cooling down to room temperature, the UV-vis absorption spectrum was collected, and the absorbance at 524 nm was recorded. A series of standard NO_2_^–^ solutions were employed to derive the calibration curve for NO_2_^–^ determination.

**Determination of H_2_ and CO**

The amounts of H_2_ and CO were quantified by a GC equipped with thermal conductivity (TCD) and flame ionization (FID) detectors. The FEs for urea, NO_2_^–^, NH_3_, CO, and H_2_ were calculated according to equations (2-6):

FE_urea_= (16 *F* × *C*_urea_ × *V*)/(60.06 × *Q*)	(2)

FE_NO2–_= (2*F* × *C*_NO2_^–^ × *V*)/(47 × *Q*)	(3)

FE_NH3_= (8*F* × *C*_NH3_ × *V*)/(17 × *Q*)	(4)

FE_CO_= (2*F* × *V/V_m_*)/*Q*	(5)

FE_H2_= (2*F* × *V/V_m_*)/*Q*	(6)

Where *F* is the Faraday constant (96500 C mol^–1^), *C* is the concentration, *V* is the volume, *V*_m_ is standard molar volume and *Q* is the total charge passed through the working electrode.

**Computational details**

Spin-polarized density functional theory (DFT) calculations were carried out using the Vienna Ab initio Simulation Package (VASP).^[S1,S2]^ Within the framework of the generalized gradient approximation (GGA), the Perdew-Burke-Ernzerhof (PBE) exchange-correlation functional was adopted, and van der Waals interactions were described via the DFT-D3 method.^[S3,S4]^ The cutoff energy for the plane-wave basis set was set to 450 eV, with an electronic energy convergence criterion of 10^–4^ eV and an atomic force convergence threshold of 0.01 eV/Å. For structural optimization, the Brillouin zone was sampled using a *k*-point grid of 1×1×1. The Ni_1_Cu_5_ and Pd_1_Cu_5_ models were based on a 12-atom Cu-based 3×1×1 supercell with a doping ratio of 1:5; Pd_1_Ni_1_Cu_5_ adopt a 16-atom Cu-based 2×2×1 supercell with a doping ratio of 1:1:5. To identify the lowest-energy structural state of Ni_1_Cu_5_, Pd_1_Cu_5_, and Pd_1_Ni_1_Cu_5_, 18 models with distinct doping sites were constructed, with 6 models investigated for each doping configuration. Then the most stable models adopted a 2×2×1 supercell, denoted as Ni_1_Cu_5_(111), Pd_1_Cu_5_(111) and Pd_1_Ni_1_Cu_5_(111), respectively, consisting of 96 atoms with six atomic layers, where the bottom two layers were fixed. A 20 Å vacuum layer was added along the z-direction to avoid interactions between periodic images.

Based on the computational hydrogen electrode model^[S5]^ proposed by Nørskov et al., the Gibbs free energy change for each elementary step can be calculated using the following formula:

$\text{Δ}\text{G}\text{ = Δ}\text{E}\text{ + ΔZPE - }\text{T}\text{Δ}\text{S}\text{ + }\int\text{C}_{\text{P}}\text{dT}$	(7)

where Δ*E*, ΔZPE, Δ*S*, and *C*_p_ were the difference of the electronic energy, the zero-point energy (ZPE), the entropy, and the heat capacity at room temperature (*T* = 298.15 K), respectively, which were calculated using a specific module in the software Vaspkit.^[S6]^ For the *R-OH and *CO intermediates, solvent effects were accounted for via stabilization energies of 0.25 eV and 0.10 eV, respectively.^[S7,S8]^

**Figure S1.** (a, b) XRD patterns of Pd_x_Ni_y_Cu_5_ ternary alloy samples with different Pd:Ni:Cu molar ratios.

**Notes:** As shown in Figure S1, the position of the diffraction pattern for (111) plane gradually shifted towards the *fcc* Pd (Figure S1a) and Ni (Figure S1b) as the Pd and Ni contents in the alloy sample gradually increased, respectively, in line with the characteristic of alloy structure. Moreover, an additional diffraction peak located at 36.5° attributed to the Cu_2+1_O arose both for Ni_1_Cu_5_ and Pd_1_Cu_5_ alloy samples. Interestingly, the peak gradually declined until totally disappeared as the Pd or Ni contents increased, suggesting that the co-doping of Pd and Ni could inhibit the oxidation of maternal copper, and improve the catalyst stability.

**Figure S2.** (a, b) TEM and amplified TEM images of Pd_1_Ni_1_Cu_5_ alloy sample. (c) The particle size distribution diagram of Pd_1_Ni_1_Cu_5_ sample.

**Figure S3.** (a-d) TEM images of the Pd_1_Cu SAAs, namely Pd_1_Cu_5_ alloy.

**Figure S4.** (a-d) EDS elemental mapping images of the Pd_1_Cu SAAs.

**Figure S5.** (a-d) TEM images of the Ni_1_Cu SAAs, namely Ni_1_Cu_5_ alloy.

**Figure S6.** (a-d) EDS elemental mapping images of the Ni_1_Cu SAAs.

**Figure S7.** (a) XPS survey spectrum and (b) Cu 2p spectrum of the Pd_1_Ni_1_Cu DAAs.

**Figure S8.** Differential charge density maps of (a) Pd_1_Cu SAAs (Pd_1_Cu_5_ alloy) and (b) Ni_1_Cu SAAs (Ni_1_Cu_5_ alloy).

**Figure S9.** The experimental (a) Pd foil and (b) Ni foil K-edge EXAFS spectra (blue line) and the fitting curves (red circle).

**Figure S10.** The experimental (a) Pd and (b) Ni K-edge EXAFS spectra (blue line) and the fitting curves (red circle) of Pd_1_Ni_1_Cu DAAs.

**Figure S11.** The corresponding EXAFS *k* space fitting curves of (a) Pd foil and (b) Ni foil.

**Figure S12.** The corresponding EXAFS *k* space fitting curves of (a) Pd and (b) Ni of Pd_1_Ni_1_Cu DAAs.

**Figure S13.** Wavelet transforms of the *k*^3^-weighted Pd and Ni K-edge EXAFS signals for the high-coordination shells with Pd foil and Ni foil.

**Figure S14.** (a) UV-vis absorption curves of diacetyl monoxime assays with varied concentrations of urea after heating at 100 °C for 20 min. (b) Working curve used for urea estimation.

**Figure S15.** (a) UV-vis absorption curves of indophenol blue assays with varied concentrations of NH_4_^+^ ions. (b) Working curve used for NH_4_^+^ estimation.

**Figure S16.** (a) UV-vis absorption curves of Griess reagent assays with varied concentrations of NO_2_**^–^** ions. (b) Working curve used for estimation of NO_2_**^–^**.

**Figure S17.** Working curves of (a) CO and (b) H_2_.

**Figure S18.** The screening of the urea electrosynthesis performance with the concentrations of nitrate.

**Figure S19.** *I*-*t* curves of electrocatalytic urea synthesis at various applied potentials over (a) Pd_1_Cu SAAs, (b) Ni_1_Cu SAAs and (c) Pd_1_Ni_1_Cu DAAs.

**Figure S20.** Comparison of urea yield rates in Ar-saturated 0.1 M KNO_3_, CO_2_-saturated 0.1 M KHCO_3_ or CO_2_-saturated 0.1 M KHCO_3_ + 0.1 M KNO_3_, respectively, over Pd_1_Ni_1_Cu DAAs at –0.5 V versus RHE.

**Figure S21.** ^1^H NMR spectra of the electrolytes with ^14^NO_3_^–^ or ^15^NO_3_^–^ as N-source in urea electrosynthesis.

**Figure S22.** ^13^C NMR spectrum of the electrolytes with ^13^CO_2_ as C-source in urea electrosynthesis.

**Notes:** As shown in Figure S21, the ^1^H NMR spectrum shows a doublet peak with chemical shift between 5.1-5.5 ppm using the ^15^NO_3_^–^ as N-feeding, the characteristic peak of CO(^15^NH_2_)_2_. While, using ^14^NO_3_^–^ as N-feeding, the characteristic signal of CO(^14^NH_2_)_2_ with a singlet peak appeared. The ^15^N-isotope labeling experiments confirmed that the urea in the electrolyte was indeed produced through the C–N coupling of CO_2_ and NO_3_^–^.^[S9]^ Moreover, the ^13^C NMR spectrum displayed the characteristic signal of ^13^CO(NH_2_)_2_, demonstrating the carbon source was CO_2_ for urea formation.^[S10]^

**Figure S23.** (a) ^1^H NMR spectra of urea with different concentrations, (b) the working curve used for urea estimation.

**Figure S24.** Comparison of quantitative results of urea electrosynthesis on Pd_1_Ni_1_Cu DAAs determined by diacetyl monoxime colorimetry, urease decomposition method and ^1^H NMR.

**Figure S25.** Potential dependence of the corresponding FEs of the possible products for Pd_1_Ni_1_Cu DAAs.

**Figure S26.** Potential dependence of (a) urea yield rates and (b) FEs of the possible by-products for Pd_1_Cu SAAs, namely Pd_1_Cu_5_ alloy.

**Figure S27.** Potential dependence of (a) urea yield rates and (b) FEs of the possible by-products for Ni_1_Cu SAAs, namely Ni_1_Cu_5_ alloy.

**Figure S28.** The comparison of urea yield rates, urea FEs and *j*_urea_ distribution for the different samples.

**Figure S29.** Cyclic voltammetry curves recorded at the different scanning rates: (a) Pd_1_Cu SAAs, (b) Ni_1_Cu SAAs, (c) Pd_1_Ni_1_Cu DAAs. (d) Comparative *C*_dl_ of those samples obtained from CV curves with different scanning rates.

**Figure S30.** Nyquist plots of the Ni_1_Cu SAAs, Pd_1_Cu SAAs and Pd_1_Ni_1_Cu DAAs samples.

**Figure S31.** The repeated durability test of urea electrosynthesis in H-cell.

**Figure S32.** XRD patterns of Pd_1_Ni_1_Cu DAAs before and after durability tests.

**Figure S33.** (a-c) TEM images, (d) HRTEM image of Pd_1_Ni_1_Cu DAAs after durability test.

**Figure S34.** (a-d) Elemental mapping images of Pd_1_Ni_1_Cu DAAs after durability test, Pd, green, Ni, blue, Cu, red.

**Figure S35.** (a) HAADF-STEM image, (b) Pd 3d and (c) Ni 2p spectra of the Pd_1_Ni_1_Cu DAAs after durability test.

**Figure S36.** (a) The changes of the pH of the electrolyte in the long-term *I-t* test and (b) XRD pattern of the dried electrolyte upon the durability test. (c) The comparison of the urea yield rates and urea FEs in the presence of K_2_CO_3_.

**Notes:** In the long-term *I-t* test in flow cell, the pH of the electrolyte remained almost at around 14. XRD pattern displayed no K_2_CO_3_ formation of the dried electrolyte upon the durability test. The analysis of the electrolyte indicated the solution of 0.76 M KOH and 0.12 M K_2_CO_3_ after the 200 h of electrolysis, delivering the theoretical pH of 13.6. We then tested the catalytic performance in the mixture of KOH + KNO_3_ + K_2_CO_3_ at –0.5 V, the urea yield rate and urea FE were comparable with that assessed without K_2_CO_3_, suggesting the produced K_2_CO_3_ did not affect the urea electrosynthesis.

**Figure S37.** (a) *I-t* curve of the metallic Cu catalyst in the co-electrolysis of CO_2_ and NO_3_^–^ at –0.5 V in the GDE. (b) The urea yield rates and urea FEs of metallic Cu catalyst assessed in the long-term *I-t* test at different times. (c) HRTEM image of metallic Cu after 5 h of durability test.

**Figure S38.** (a) The potential dependence of the electrochemical activity in the sole CO_2_RR-to-CO and (b) the sole NO_3_RR-to-NH_3_ conversions over Pd_1_Cu SAAs, Ni_1_Cu SAAs and Pd_1_Ni_1_Cu DAAs.

**Figure S39.** The potential dependence of (a) CO FEs in the sole CO_2_RR and (b) NH_3_ FEs in the sole NO_3_RR for Pd_1_Cu SAAs, Ni_1_Cu SAAs and Pd_1_Ni_1_Cu DAAs.

**Figure S40.** Potential dependence of (a) *j*_CO/ECSA_ in sole CO_2_RR and (b) *j*_NH3/ECSA_ in sole NO_3_RR of Pd_1_Ni_1_Cu DAAs, Ni_1_Cu SAAs and Pd_1_Cu SAAs normalized to ECSA.

**Figure S41.** Comparison of the urea yield rates and urea FEs of Pd_1_Cu SAAs, Ni_1_Cu SAAs, a physical mixture of Pd_1_Cu SAAs and Ni_1_Cu SAAs, and the Pd_1_Ni_1_Cu DAAs samples.

**Figure S42.** ELF picture of Ni_1_Cu DAAs.

**Figure S43.** Differential charge density maps of PdCu sites with the single-atom Ni_1_ site (a) far away from the PdCu site or (b) located in the middle of PdCu site.

**Notes:** Bader charge analysis indicates that Pd and Ni atoms obtain –0.43 and –0.06 *e*^–^, respectively, while Cu atom loses 0.06 *e*^–^ for the Ni_1_ adjacent to the PdCu site. For the case of Ni_1_ away from PdCu site, Pd and Ni atoms obtain –0.36 and –0.08 *e*^–^, respectively, and Cu loses 0.02 *e*^–^, demonstrating that Ni_1_ acts as the electron bridge to facilitate the charge transfer from Cu to Pd site.

**Figure S44.** PDOS profiles of Pd_1_Cu SAAs, Ni_1_Cu SAAs and Pd_1_Ni_1_Cu DAAs.

**Figure S45.** DFT-calculated urea formation cycle on Pd_1_Cu (111) SAAs surface (side view).

**Figure S46.** DFT-calculated urea formation cycle on Ni_1_Cu(111) SAAs surface (side view).

**Figure S47.** ELF pictures of *CO on Ni_1_Cu(111) SAAs and Pd_1_Ni_1_Cu(111) DAAs.

**Table S1.** Molar ratios of Pd, Ni, Cu in the Pd_x_Ni_y_Cu_5_ samples determined by ICP-AES.

| Catalyst | Molar ratio of Pd:Ni:Cu |
| --- | --- |
| Pd_0.1_Ni_1_Cu_5_ | 1.2:9.0:89.8 |
| Pd_1_Ni_1_Cu_5_ | 10.0:10.0:80.0 |
| Pd_2_Ni_1_Cu_5_ | 18.0:10.0:72.0 |
| Pd_5_Ni_1_Cu_5_ | 45.0:9.0:46.0 |
| Pd_1_Ni_0.1_Cu_5_ | 11.0:1.3:87.7 |
| Pd_1_Ni_0.5_Cu_5_ | 10.0:6.8:83.2 |
| Pd_1_Ni_2_Cu_5_ | 13.0:19.0:68.0 |

**Table S2.** EXAFS fitting parameters of the Pd and Ni K-edge for various samples (*Ѕ*_0_^2^= 1.0).

| Sample | Path | *N*^a^ | *R*(Å)^b^ | *σ*^2^(Å^2^)^c^ | Δ*E*_0_(eV)^d^ | *R* factor |
| --- | --- | --- | --- | --- | --- | --- |
| Pd foil | Pd-Pd | 12 | 2.74 | 0.005 | 4.498±0.31 | 0.008 |
| Ni foil | Ni-Ni | 12 | 2.49 | 0.006 | 6.457±0.84 | 0.006 |
| Sample Pd | Pd-Cu | 7.26 | 2.72 | 0.005 | 2.297±0.25 | 0.005 |
| Sample Ni | Ni-Cu | 6.73 | 2.54 | 0.008 | 3.486±0.88 | 0.011 |
|  | Ni-O | 3.07 | 1.96 | 0.006 | 6.552±0.59 | 0.009 |

**Table S3.** Urea electrosynthesis from C–N coupling in comparison with the recently reported state-of-the-art electrocatalysts in H-cell.

| **Catalyst** | **N/C sources** | **Electrolyte** | **Urea yield rate (mmol h^–1^ g_cat._^–1^)** | **FE_urea_**  **(%)** | **Potential (V vs. RHE)** | **Refs.** |
| --- | --- | --- | --- | --- | --- | --- |
| m-Cu_2_O | NO_3_^–^+CO_2_ | 0.1 M KNO_3_+  0.1 M KHCO_3_ | 29.2 | 9.43 | –1.3 | S11 |
| Fe–Ni | NO_3_^–^+CO_2_ | 0.05 M KNO_3_+  0.1 M KHCO_3_ | 20.2 | 17.8 | –1.5 | S12 |
| Cu SACs | NO_3_^–^+CO_2_ | 0.1 M KNO_3_+ 0.1 M KHCO_3_ | 30.0 | 28 | –0.9 | S13 |
| Fe(a)@C  –Fe_3_O_4_/CNTs | NO_3_^–^+CO_2_ | 0.1 M KNO_3_ | 22.33 | 16.5 | –0.65 | S14 |
| MoO_x_/C | NO_3_^–^+CO_2_ | 0.1 M KNO_3_ | 23.83 | 27.7 | –0.6 | S15 |
| FeN_3_ | NO_3_^–^+CO_2_ | 0.1 M KNO_3_ | 8.27 | 16.6 | –0.9 | S16 |
| F-CNT | NO_3_^–^+CO_2_ | 0.1 M KNO_3_ | 6.36 | 18 | –0.65 | S17 |
| Ag-CuNi(  OH)_2_-0.3:5 | NO_3_^–^+CO_2_ | 0.1 M KNO_3_+ 0.1 M KHCO_3_ | 25.6 | 46.1 | –0.5 | S18 |
| Cu-MoSe_2_ | NO_3_^–^+CO_2_ | 0.1 M KNO_3_ | 20.6 | 23.4 | –0.7 | S19 |
| Cu@Fe-N-C | NO_2_^–^+CO_2_ | 100 ppm (NO_2_^–^ -N, ~0.007 M for NO_2_^–^) | 14.2 | 50.1 | –0.45 | S20 |
| ZnO-V | NO_2_^–^+CO_2_ | 0.2 M NaHCO_3_ + 0.1 M NaNO_2_ | 16.56 | 23.26 | –0.79 | S21 |
| Cu_97_In_3_-C | NO_3_^–^+CO_2_ | 0.1 M KHCO_3_ + 0.01 M KNO_3_ | 13.1 | / | –1.4 | S22 |
| XC72R-AuPd | NO_3_^–^+CO_2_ | 0.075 M KHCO_3_ + 0.025 M KNO_3_ | 3.4 | 15.6 | –0.5 | S23 |
| **Pd_1_Ni_1_Cu**  **DAAs** | **NO_3_^–^+CO_2_** | **0.1 M KNO_3_+ 0.1 M KHCO_3_** | **78.8** | **35.0** | **–0.5** | **This work** |

**Table S4.** Urea electrosynthesis from C–N coupling in comparison with the recent state-of-the-art electrocatalysts in flow cell.

| **Catalyst** | **N/C sources** | **Electrolyte** | **Urea yield rate** | **FE_urea_**  **(%)** | **Potential**  **(V vs. RHE)** | **Stability (h)** | **Refs.** |
| --- | --- | --- | --- | --- | --- | --- | --- |
| CuSiO_x_ | NO_3_^–^+CO_2_ | 0.1 M KNO_3_+  0.1 M KHCO_3_ | 26.7 | 79 | –0.6 | 80 | S24 |
| Ag_67_Cu_33_ | NO_3_^–^+CO_2_ | 0.5 M KNO_3_+  0.5 M KHCO_3_ | 54.8 | 36.6 | –0.52 | 24 | S25 |
| SnO_2_/CuO_x_ | NO_3_^–^+CO_2_ | 0.1 M KNO_3_+ 0.1 M KHCO_3_ | 215 | 71.2 | –0.5 | 240 | S26 |
| PdCuCo | NO_3_^–^+CO_2_ | 0.5 M KNO_3_+  0.5 M KHCO_3_ | 227 | 81 | –0.6 | 260 | S10 |
| CuRu/MCHS | NO_3_^–^+CO_2_ | 0.1 M KNO_3_ | 58.3 | 16.5 | –0.7 | 125 | S27 |
| PdH_x_/Cu | NO_3_^–^+CO_2_ | 1.0 M KOH +  0.1 M KNO_3_ | 237 | 62.6 | –0.7 | 200 | S28 |
| CuPd_1_Rh_1_ | NO_3_^–^+CO_2_ | 0.1 M KNO_3_+ 0.1 M KHCO_3_ | 53.2 | 72.1 | –0.5 | 100 | S29 |
| Ag_0.07_/CdO | NO_3_^–^+CO_2_ | 1.0 M KOH +  0.05 M KNO_3_ | 112 | 50.0 | –0.15 | 1000 | S30 |
| CuSn/CS | NO_3_^–^+CO_2_ | 0.1 M KNO_3_ | 155 | 74.7 | –0.7 | 10 | S31 |
| Cu_3_Mo_2_O_9_ | NO_3_^–^+CO_2_ | 0.1 M KNO_3_ | 177 | 40 | –0.8 | 20 | S32 |
| **Pd_1_N_1_Cu**  **DAAs** | **NO_3_^–^+CO_2_** | **1.0 M KOH +0.1 M KNO_3_** | **541.6** | **57.3** | **–0.5** | **1700** | **This work** |

**Note:** The unit for urea yield rate is mmol g_cat._^–1^ h^–1^, respectively.

**Table S5.** Control experiments to decode C- and N-intermediates for C–N coupling at –0.5 V using Pd_1_Ni_1_Cu DAAs as a catalyst.

| Entry | C-source | N-source | Electrolyte solution | Urea yield rate (mmol g_cat_^–1^ h^–1^) | Urea FE  (%) |
| --- | --- | --- | --- | --- | --- |
| 1 | CO_2_ | KNO_3_ | 100 mM KHCO_3_+ 100 mM KNO_3_ | 78.8 | 35.0 |
| 2 | CO | KNO_3_ | 100 mM KNO_3_ | 37.1 | 11.9 |
| 3 | HCHO | KNO_3_ | 100 mM KNO_3_+20 mM HCHO | N.A. | N.A. |
| 4 | HCOOH | KNO_3_ | 100 mM KNO_3_+20 mM HCOOH | 43.0 | 18.4 |
| 5 | CH_3_OH | KNO_3_ | 100 mM KNO_3_+20 mM CH_3_OH | N.A. | N.A. |
| 6 | KHCO_3_+CO_2_ | NH_3_ | 100 mM KHCO_3_+20 mM NH_3_ | N.A. | N.A. |
| 7 | KHCO_3_+CO_2_ | NH_4_Cl | 100 mM KHCO_3_+20 mM NH_4_Cl | N.A. | N.A. |
| 8 | KHCO_3_+CO_2_ | HCONH_2_ | 100 mM KHCO_3_+20 mM HCONH_2_ | 12.5 | 10.0 |
| 9 | KHCO_3_+CO_2_ | KNO_2_ | 100 mM KHCO_3_ +100 mM KNO_2_ | 32.7 | 26.6 |
| 10 | KHCO_3_+CO_2_ | NH_2_OH | 100 mM KHCO_3_+20 mM NH_2_OH | 21.2 | 14.9 |

**Table S6.** Bader charge analysis of Pd, Ni, Cu atoms in Pd_1_Ni_1_Cu DAAs, Pd_1_Cu SAAs and Ni_1_Cu SAAs for the models in Figures 5a, S42, –denotes obtaining electron, + denotes losing electron.

| Sample | Cu atom | Pd atom | Ni atom | Sum atom |
| --- | --- | --- | --- | --- |
| Pd_1_Cu SAAs | 0.47 e^–^ | –1.14 e^–^ | / | –0.66 e^–^ |
| Ni_1_Cu SAAs | 0.03 e^–^ | / | –0.26 e^–^ | –0.23 e^–^ |
| Pd_1_Ni_1_Cu DAAs | 0.30 e^–^ | –1.15 e^–^ | –0.16 e^–^ | –1.01 e^–^ |

**Reference:**

S1. Kresse, G.; Furthmüller, J. Efficient iterative schemes for abinitio total-energy calculations using a plane-wave basis set, *Phys. Rev. B.* **1996**, *54*, 11169−11186.

S2. Kresse G, Hafner J. Ab initio molecular dynamics for liquid metals, *Phys. Rev. B* **1993**, *47*, 558-561.

S3. Perdew, J. P.; Burke, K.; Ernzerhof, M. Generalized gradient approximation made simple, *Phys. Rev. Lett.* **1996**, *77*, 3865−3868.

S4. Blöchl, P. E.; Först, C. J.; Schimpl, J. Projector augmented wave method: ab initio molecular dynamics with full wave functions, *Bulletin of Mater. Sci.* **2003**, *26*, 33−41.

S5. Nørskov, J. K.; Rossmeisl, J.; Logadottir, A. et al. Origin of the overpotential for oxygen reduction at a fuel-cell cathode, *J. Phys. Chem. B* **2004**, *108*, 17886−17892.

S6. Wang, V.; Xu, N.; Liu, J. et al. VASPKIT: a user-friendly interface facilitating high-throughput computing and analysis using VASP code, *Comp. Phys. Commun.* **2021**, *267*, 108033.

S7. Bai, X. X.; Zhao, Z. L.; Lu, G. Breaking the scaling relationship on single-atom embedded MBene for selective CO_2_ electroreduction, *J. Phys. Chem. Lett.* **2023**, *14*, 5172–5180.

S8. Li, Y. W.; Su, H. B.; Chan, S. H. et al. CO_2_ electroreduction performance of transition metal dimers supported on graphene: a theoretical study, *ACS Catal.* **2015**, *5*, 6658−6664.

S9. Yuan, M.; Chen, J.; Xu, Y.; Liu, R.; Zhao, T.; Zhang, J.; Ren, Z.; Liu, Z.; Streb, C.; He, H.; Yang, C.; Zhang, S.; Zhang, G. Highly selective electroreduction of N_2_ and CO_2_ to urea over artificial frustrated Lewis pairs, *Energy Environ. Sci.* **2021**, *14*, 6605–6615.

S10. M. Xu, H. Zhou, X. Lv, Y. Fang, X. Tu, F. Wang, Q. Han, X. Wang, G. Zheng, Selective urea electrosynthesis from CO_2_ and nitrate on spin‐polarized atomically ordered PdCuCo, *Adv. Mater.* **2025**, *37*, 2505286.

S11. M. Qiu, X. Zhu, S. Bo, K. Cheng, N. He, K. Gu, D. Song, C. Chen, X. Wei, D. Wang, Y. Liu, S. Li, X. Tu, Y. Li, Q. Liu, C. Li, S. Wang, Boosting electrocatalytic urea production via promoting asymmetric C–N coupling, *CCS Chem.* **2023**, *5*, 2617−2627.

S12. X. Zhang, X. Zhu, S. Bo, C. Chen, M. Qiu, X. Wei, N. He, C. Xie, W. Chen, J. Zheng, P. Chen, S. P. Jiang, Y. Li, Q. Liu, S. Wang. Identifying and tailoring C–N coupling site for efficient urea synthesis over diatomic Fe–Ni catalyst, *Nat. Commun.* **2022**, *13*, 5337.

S13. J. Leverett, T. Tran-Phu, J. A. Yuwono, P. Kumar, C. Kim, Q. Zhai, C. Han, J. Qu, J. Cairney, A. N. Simonov, R. K. Hocking, L. Dai, R. Daiyan, R. Amal, Tuning the coordination structure of Cu-N-C single atom catalysts for simultaneous electrochemical reduction of CO_2_ and NO_3_^–^ to urea, *Adv. Energy Mater.* **2022**, *12*, 2201500.

S14. J. Geng, S. Ji, M. Jin, C. Zhang, M. Xu, G. Wang, C. Liang, H. Zhang, Ambient electrosynthesis of urea with nitrate and carbon dioxide over iron‐based dual‐sites, *Angew. Chem. Int. Ed*. **2023**, *62*, e202210958.

S15. M. Sun, G. Wu, J. Jiang, Y. Yang, A. Du, L. Dai, X. Mao, Q. Qin, Carbon‐anchored molybdenum oxide nanoclusters as efficient catalysts for the electrosynthesis of ammonia and urea, *Angew. Chem. Int. Ed*. **2023**, *62*, e202301957.

S16. T. Hou, J. Ding, H. Zhang, S. Chen, Q. Liu, J. Luo, X. Liu. FeNi_3_ nanoparticles for electrocatalytic synthesis of urea from carbon dioxide and nitrate, *Mater. Chem. Front.* **2023**, *7*, 4952–4960.

S17. X. Liu, P. V. Kumar, Q. Chen, L. Zhao, F. Ye, X. Ma, D. Liu, X. Chen, L. Dai, C. Hu, Carbon nanotubes with fluorine-rich surface as metal-free electrocatalyst for effective synthesis of urea from nitrate and CO_2_, *Appl. Catal. B* Environ. **2022**, *316*, 121618.

S18. W. Ye, Y. Zhang, L. Chen, F. Wu, Y. Yao, W. Wang, G. Zhu, G. Jia, Z. Bai, S. Dou, P. Gao, N. Wang. G. Wang, A strongly coupled metal/hydroxide heterostructure cascades carbon dioxide and nitrate reduction reactions toward efficient urea electrosynthesis, *Angew. Chem. Int. Ed*. **2024**, *63*, e202410105.

S19. J. Jiang, G. Wu, M. Sun, Y. Liu, Y. Yang, A. Du, L. Dai, X. Mao, Q. Qin, Cu–Mo dual sites in Cu-Doped MoSe_2_ for enhanced electrosynthesis of urea, *ACS Nano* **2024**, *18*, 13745–13754.

S20. Z. Feng, L. Zhang, Y. Guo, J. Guo, F. Li, F. Yu, Charge polarization boosting electrochemical urea synthesis by co-reduction of CO_2_ and nitrite in dilute concentrations with a unity carbon selectivity, *Angew. Chem. Int. Ed.* **2025**, e202500262.

S21. N. Meng, Y. Huang, Y. Liu, Y. Yu, B. Zhang, Electrosynthesis of urea from nitrite and CO_2_ over oxygen vacancy-rich ZnO porous nanosheets, *Cell Rep. Phy. Sci.* **2021**, *2*, 100378.

S22. Y. Liu, X. Tu, X. Wei, D. Wang, X. Zhang, W. Chen, C. Chen, S. Wang, C‐bound or O‐bound surface: which one boosts electrocatalytic urea synthesis? *Angew. Chem. Int. Ed*. **2023**, *62*, e202300387.

S23. H. Wang, Y. Jiang, S. Li, F. Gou, X. Liu, Y. Jiang, W. Luo, W. Shen, R. He, M. Li, Realizing efficient C-N coupling via electrochemical co-reduction of CO_2_ and NO_3_^-^ on AuPd nanoalloy to form urea: Key C-N coupling intermediates, *Appl. Catal. B* Environ. **2022**, *318*, 121819.

S24. W. Qiu, S. Qin, Y. Li, N. Cao, W. Cui, Z. Zhang, Z. Zhuang, D. Wang, Y. Zhang, Overcoming electrostatic interaction via pulsed electroreduction for boosting the electrocatalytic urea synthesis, *Angew. Chem., Int. Ed.* **2024**, *63*, e202402684.

S25. P. Feng, S. Wang, Z. Dai, Y. Chen, B. Zhang, M. Cheng, B. Ke, J. Xia, G. Zhang, F. Huang, Stable Cu^δ+^ sites derived from analogous rectifying interface in AgCu biphasic aerogels for efficient urea electrosynthesis at low potential, *Nat. Commun.* **2026**, *17*, 59.

S26. H. Cheng, R. Ma, S. Liu, K. Li, C. Chen, R. Gui, J. Peng, M. Zhou, W. Zhang, W. Bi, W. Chu, G. Wang, C. Xiao, Y. Xie, C. Wu, Enriching local reaction fields via ordered multidimensional interfaces for high-yield urea electrosynthesis, *J. Am. Chem. Soc*. **2026**, *148*, 8812–8822.

S27. J. Du, Y. Wu, S. Fang, D. Xu, M. Liu, H. Liang, Z. Wu, G. M. Lu, X. Wu, Nano-confinement engineering boosts C–N coupling for urea electrosynthesis, *Nat. Commun.* **2025**, *17*, 1008.

S28. Y. Li, B. Han, Y. Liu, Y. Liu, R. Zhang, B. Wang, Y. Chen, B. Y. Xia, J. Li, Dual spillover of carbon monoxide and hydrogen initiates tandem urea electrosynthesis, *Nat. Commun.* **2026**, *17*, 2506.

S29. K. Chen, D. Ma, Y. Zhang, F. Wang, X. Yang, X. Wang, H. Zhang, X. Liu, R. Bao, K. Chu, Urea electrosynthesis from nitrate and CO_2_ on diatomic alloys, *Adv. Mater.* **2024**, *36*, 2402160.

S30. S. Liu, T. Wang, J. Liu, J. Liu, H. Shi, Z. Lin, Z. Cai, Y. Huang, Q. Li, Selective electrosynthesis of urea from nitrate and carbon dioxide with low overpotential, *Nat. Commun.* **2026**, *17*, 1787.

S31. X. Wu, Y. Chen, B. Tang, Q. Yan, D. Wu, H. Zhou, H. Wang, H. Zhang, D. He, H. Li, J. Zeng, L. Lu, S. Yang, T. Ma, CeO_x_-integrated dual site enhanced urea electrosynthesis from nitrate and carbon dioxide, *Nat. Commun.* **2025**, *16*, 8785.

S32. C. Zhang, Q. Zhou, Z. Li, C. Yan, H. Liu, D. Liu, L. Song, Q. Yan, C. Lv, Promoting the intermediates hydrogenation for urea electrosynthesis over an ‘active hydrogen pump’ catalyst, *Angew. Chem. Int. Ed.* **2025**, *64*, e202507869.
